# Factors influencing identification accuracy rate of parathyroid glands using near-infrared autofluorescence during endoscopic thyroidectomy

**DOI:** 10.1016/j.sopen.2026.08.001

**Published:** 2026-08-10

**Authors:** Lihua Ao, Yilin Li, Jiaqi Cen, Huiyuan Tang, Wanyu Sun, Zifeng Luo, Chang Cai, Song Wang

**Affiliations:** a Nanshan School, Guangzhou Medical University, Guangzhou, Guangdong, China; b School of Languages and Cultures, Hunan Institute of Technology, Hengyang, Hunan, China; c Department of Surgery Teaching and Research, the Fifth Clinical College, Guangzhou Medical University, Guangzhou, China; d Department of General Surgery, Guangdong Engineering Technology Research Center of Biological Targeting Diagnosis, Therapy and Rehabilitation, The Fifth Affiliated Hospital of Guangzhou Medical University, Guangzhou, China

**Keywords:** False negative, False positive, Near-infrared autofluorescence, Parathyroid glands, Thyroidectomy

## Abstract

**Background:**

Near-infrared autofluorescence (NIRAF) enhances parathyroid gland (PG) identification during thyroidectomy. However, its identification rate is frequently affected by false-positive and false-negative results.

**Methods:**

This prospective cohort study enrolled 50 patients scheduled for endoscopic thyroidectomy, employing a 1:1 case-matched design. The patients were assigned to an experimental group (NIRAF group; *n* = 25) and a control group (Non-NIRAF group; n = 25).

**Results:**

In the NIRAF group, levels of parathyroid hormone (PTH) on the first postoperative day (*P* = 0.002) and at the first postoperative month (*P* = 0.04), as well as serum calcium levels at the first month, were significantly higher than those in the Non-NIRAF group (*P* = 0.03). The sensitivity and specificity of NIRAF for identifying PGs were 80% and 84%, respectively. A higher body mass index (BMI) was associated with an increased incidence of false negatives (*P* = 0.07). Four false-positive events were recorded, originating from a thyroid nodule, thymus, adipose tissue, and a lymph node.

**Conclusion:**

NIRAF is a highly sensitive navigation tool that effectively improves PG preservation and postoperative outcomes. Nevertheless, its diagnostic accuracy is challenged by false positives from heterogeneous tissues and false negatives driven by anatomical barriers like excessive fat.

## Graphical abstract

**Figure:**
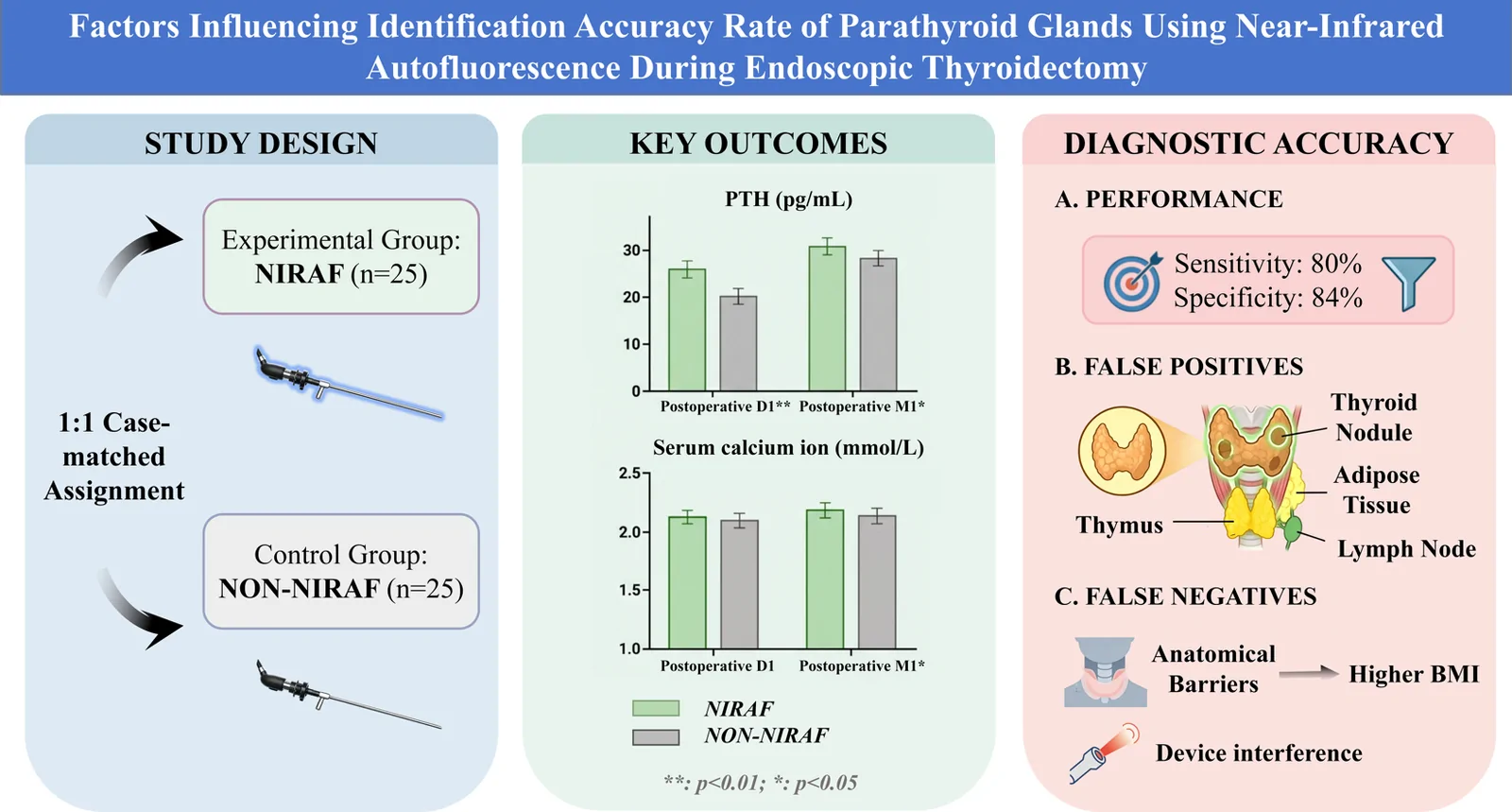

## Introduction

Thyroid cancer accounts for approximately 2.2% of all cancer cases worldwide, and surgical resection is the most important treatment modality [1], [2]. The parathyroid glands (PGs) are located adjacent to the thyroid gland. Exhibiting considerable anatomical variability, the PGs usually weigh 35–45 mg, and measure 5 mm × 3 mm × 1 mm [3]. Because of their small size and vulnerable vascularization, the identification accuracy and preservation of the PGs remain challenging during thyroidectomy. PGs can be inadvertently removed, which leads to postoperative complications, such as hypoparathyroidism and hypocalcemia, affecting patients' long-term quality of life [4], [5]. According to the guidelines of the American Thyroid Association, the incidence of hypoparathyroidism after total thyroidectomy ranges from 23.6% in transient cases to 6.5% in permanent cases [6]. However, conventional strategies for intraoperative identification, including visual inspection, intraoperative frozen section analysis, and the use of lateral flow strips to detect parathyroid hormone (PTH) in fine-needle aspirations, are often limited by subjectivity and time delay. Therefore, novel technologies are being actively explored to improve this situation, with near-infrared autofluorescence (NIRAF) emerging as a novel intraoperative technology aimed at enhancing the visualization of PGs during thyroid surgery [7], [8].

NIRAF is a highly anticipated technology that exploits the unique autofluorescent properties of parathyroid tissue under near-infrared light [9]. Autofluorescence is a process in which biomolecules and tissues are excited by near-infrared light [10]. As reported by Kiernan CM et al., when the excitation light is at 785nm, the PG emits a distinct autofluorescence signal at 820–830 nm, with an intensity 5 to 12 times higher than that of the surrounding tissues [11], [12], [13], [14], [15]. Numerous randomized controlled trials and meta-analyses have confirmed the clinical efficacy of NIRAF, demonstrating a substantial improvement in PG identification rate and a significant reduction in the incidence of transient hypocalcemia from 21.7% to 9.1%, but these studies have not revealed any statistically significant differences in permanent hypoparathyroidism [13], [16], [17].

The identification rate of NIRAF is affected by numerous objective factors, such as false-positive and false-negative interpretations, which fail to meet the expectations of clinicians [8], [18]. For instance, a recent study reported a false-positive rate of 32.1% in parathyroid identification [14]. Similarly, in a study of 19 pediatric patients, it was shown that at a lower threshold (≥1.2), the false-positive rate reached 21.6% [15]. Conversely, false-negative results can also occur. A study showed that NIRAF failed to identify 8 of 264 (3%) glands [19]. There are numerous factors contributing to those false positives and false negatives results. However, the primary cause remains unclear [7], [15].

Therefore, a comprehensive analysis of the specific sources of the errors and their contributing factors is necessary. In view of these critical limitations, this study systematically analyzed the specific factors that affect the accuracy of NIRAF in endoscopic thyroidectomy. Through a comprehensive exploration of these challenges, our research aims to contribute to the development of multimodal verification strategies that can sustainably improve both the discriminatory accuracy and overall safety of surgeries using NIRAF.

## Methods

### Study design

This prospective cohort study enrolled 50 patients scheduled for endoscopic thyroidectomy between March 2023 and March 2025. To minimize selection bias, a predefined 1:1 case-matched design was employed. The patients were assigned to the experimental group (NIRAF group; *n* = 25) to receive intraoperative NIRAF assistance, or the control group (Non-NIRAF group; n = 25) to undergo the procedure without NIRAF. Patients were prospectively matched based on baseline demographic characteristics and biochemical markers. This study was approved by the Ethics Review Committee (GYWY-L2023-77). Written informed consent was obtained from all patients prior to the enrollment.

### Participants

We screened patients aged ≥18 years who were scheduled to undergo an initial endoscopic thyroidectomy. Patients were included if they had no contraindications to general anesthesia and agreed to comply with the scheduled follow-up protocol. Patients were excluded prior to enrollment based on the following criteria: (1) preexisting parathyroid disease or abnormal baseline calcium/PTH levels; (2) recent use of medications known to affect calcium or PTH metabolism (e.g., bisphosphonates, thiazide diuretics, lithium) within 3 months prior to surgery; (3) history of prior thyroid surgery, neck surgery, or neck irradiation; (4) severe liver or kidney dysfunction; (5) history of other malignancies or active antitumor therapy; (6) pregnancy; and (7) inability to cooperate owing to language barriers. Patients who required intraoperative conversion to open thyroidectomy were excluded from the final analysis.

### Techniques

All procedures were performed using 4K ultra-high-definition endoscopic systems from the same manufacturer (OptoMedic, China; OPTO-CAM214K). This system allows the surgeon to shift among three intraoperative visualization modes: standard white-light, NIRAF and a fusion mode.

### Surgery

All surgeries were performed by the same team of senior physicians with >16 years of experience in thyroidectomy.

#### Non-NIRAF group

Patients assigned to this group underwent standard endoscopic thyroidectomy under general anesthesia. After establishing the workspace, meticulous capsular dissection was performed to safely ligate the vessels and completely mobilize the thyroid gland. During excision and central lymph node dissection, the surgeon relied on the standard endoscopic white-light mode and anatomical landmarks to identify and preserve the PGs in situ. The excised specimens were meticulously inspected ex vivo to ensure that no PGs were inadvertently removed.

#### NIRAF group

The foundational surgical procedures were identical to those in the Non-NIRAF group. To facilitate PG localization, the NIRAF mode was activated. Systematic NIRAF scanning of the surgical field was continuously performed before, during, and after capsular dissection to identify and preserve PGs in situ. Furthermore, all excised specimens were routinely examined ex vivo using NIRAF. If NIRAF detected any suspected PG within the excised tissue, immediate parathyroid autotransplantation was performed.

### Data collection

Baseline patient characteristics, preoperative assessments, and specific surgical details were prospectively collected. Serum calcium and intact parathyroid hormone (PTH) levels were measured preoperatively, on postoperative day one, and during scheduled follow-up visits. Postoperative hypoparathyroidism and biochemical hypocalcemia were defined as intact PTH levels <15 pg/mL and serum calcium concentrations <2.10 mmol/L [6], [20]. All surgical procedures were continuously video recorded to extract and analyze specific NIRAF imaging parameters and autofluorescence characteristics (e.g., brightness, uniformity, and edge definition) of both true PGs and confounding false-positive tissues. Inadvertent parathyroidectomy was documented if parathyroid tissue was histopathologically identified in any of the resected specimens.

### Statistical analysis

All enrolled participants were included in the analysis as prespecified. Missing data were not imputed. Continuous outcomes were summarized as mean ± SD if approximately normally distributed and compared using two sample *t-*tests; otherwise, outcomes were summarized as medians (minimum–maximum) and analyzed using the Mann-Whitney *U* test. Changes in PTH and serum calcium levels were analyzed across pre-specified time points, and between-group comparisons were performed at corresponding visits. To evaluate the diagnostic performance of NIRAF, sensitivity and specificity were calculated. Furthermore, univariate and multivariate logistic regression models were prospectively planned and employed to identify the association between baseline patient factors (such as BMI, age, and sex) and the risk of inadvertent parathyroidectomy. A two-sided *p*-value <0.05 was considered statistically significant.

## Results

### Characteristics of patients

Fifty patients who met the inclusion criteria were prospectively enrolled in this study and assigned to either the NIRAF group (*n* = 25) or the Non-NIRAF group (n = 25). There were no statistically significant differences between the two groups in terms of baseline data (*P* > 0.05) (Table 1).

**Table 1:** Comparison of general patient data.

	NIRAF group (*n* = 25)	Non-NIRAF group (n = 25)	*P* value
Sex, n (%)			0.19
Male	8 (32.0)	4 (16.0)	
Female	17 (68.0)	21 (84.0)	
Age (year), median (IQR)	37.00 (29.00, 50.50)	43.00 (35.50, 47.00)	0.32
BMI (kg/m^2^), median (IQR)	23.28 (21.03, 25.61)	24.34 (21.70, 26.77)	0.53
Preoperative PTH (pg/ml), mean ± SD	35.40 ± 14.24	34.89 ± 10.00	0.88
Preoperative serum calcium (mmol/L), median (IQR)	2.28 (2.22, 2.35)	2.28 (2.25, 2.36)	0.79

Abbreviations: BMI: body mass index; IQR, interquartile range; NIRAF, near-infrared autofluorescence; PTH: parathyroid hormone; SD, standard deviation.

### Postoperative outcomes

The comparison of biochemical markers after surgery was significant. Compared with the non-NIRAF group, PTH levels in the NIRAF group were significantly higher (*P* < 0.05) (Table 2). Precisely, the NIRAF group was 6 pg/mL higher on the first day after surgery and 2.47 pg/mL higher on the first month after surgery. Serum calcium levels showed no significant difference between the two groups on the first postoperative day (*P* > 0.05). However, the median (interquartile range) in the NIRAF group [2.18 (2.13, 2.35) mmol/L] was significantly higher than that in the Non-NIRAF group [2.13 (2.09, 2.18) mmol/L] on the first postoperative month (*P* < 0.05) (Table 2).

**Table 2:** Comparison of operative and postoperative outcomes.

	NIRAF Group (n = 25)	Non-NIRAF Group (n = 25)	P value
Resection Scope			0.77
Unilateral resection, n (%)	14 (56.0)	15 (60.0)	
Bilateral resection, n (%)	11 (44.0)	10 (40.0)	
Surgical Safety			0.33
Inadvertent parathyroidectomy, n (%)	5 (20.0)	8 (32.0)	
Sensitivity	80.0	68.0	
Specificity	84.0	NA	
Postoperative Function			
PTH (pg/mL)			
Postoperative D1, median (IQR)	26.20 (20.50, 33.16)	20.20 (14.60, 24.25)	0.002
Postoperative M1, median (IQR)	30.90 (26.50, 44.55)	28.43 (25.57, 30.42)	0.04
Serum calcium ion (mmol/L)			
Postoperative D1, mean ± SD	2.12 ± 0.11	2.08 ± 0.15	0.29
Postoperative M1, median (IQR)	2.18 (2.13, 2.35)	2.13 (2.09, 2.18)	0.03

Abbreviations: D1, the first day; IQR, interquartile range; M1, the first month; NIRAF, near-infrared autofluorescence; PTH: parathyroid hormone; SD, standard deviation.

### Inadvertent parathyroidectomy events

In the NIRAF group, the sensitivity of parathyroid identification was 80%, and the specificity was 84%. Of all these patients, eight inadvertent parathyroidectomy events occurred in the Non-NIRAF group, whereas five occurred in the NIRAF group (Table 2). Although the difference between the two groups was not statistically significant (*P* > 0.05), the NIRAF group demonstrated a lower rate of parathyroid misidentification.

### Impact of BMI on inadvertent parathyroidectomy

Regarding the relationship between baseline patient factors and the risk of inadvertent parathyroidectomy, univariate binary logistic regression demonstrated a significant association between higher BMI and the occurrence of inadvertent parathyroidectomy (*P* = 0.04, 95% CI: 1.01–1.76). However, after adjusting for potential confounders, including age and sex, in the multivariate binomial logistic regression analysis, this association did not strictly reach statistical significance (*P* = 0.07, 95% CI: 0.98–1.73).

### False-positive events in NIRAF group

**Fig. 1:**
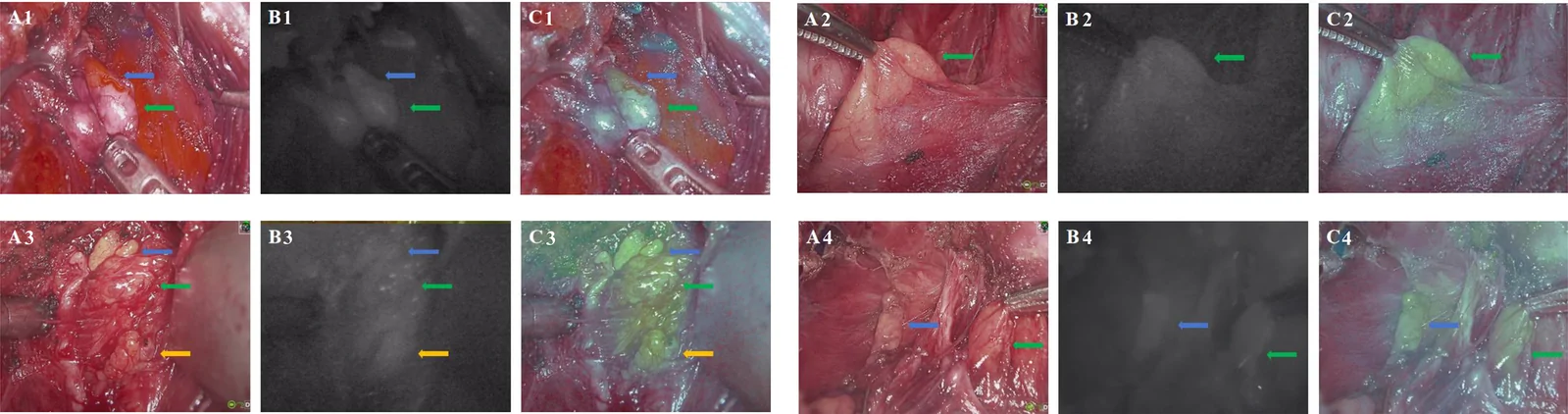
Representative intraoperative endoscopic images of false-positive autofluorescence in the NIRAF group. Images are displayed in white-light (A1–A4), NIRAF (B1–B4), and fusion (C1–C4) modes. (A1–C1) Residual thyroid adenoma (green arrow) adjacent to the right superior PG (blue arrow). (A2–C2) Thymic tissue (green arrow). (A3–C3) Left adipose mass (green arrow) visualized alongside the left superior PG (blue arrow) and left inferior PG (orange arrow). (A4–C4) Lymph node in level VIB (green arrow) near the right inferior PG (blue arrow). (For interpretation of the references to color in this figure legend, the reader is referred to the web version of this article.)

Table 3 summarizes the four false-positive events observed in the NIRAF group. Case 1 represents a typical false-positive instance, in which a thyroid nodule exhibited autofluorescence mimicking PGs (Fig. 1, A1–C1). In case 3, a suspected autofluorescent tissue was detected and confirmed by the pathology to be adipose tissue rather than PGs (Fig. 1, A3–C3). Similarly, other non-parathyroid tissues, such as the thymus (case 2) and lymph nodes (case 4), were also found to emit confounding autofluorescent signals (Fig. 1, A2–C2, A4–C4). Intraoperatively, these suspected fluorescent tissues were excised, separately labelled, and submitted for pathological examination, which ultimately confirmed that they were not parathyroid. Furthermore, no residual parathyroid tissue was found in the primary surgical specimens. No actual PGs were inadvertently removed. All four patients maintained normal parathyroid function. As shown in Table 3, their one-month postoperative PTH and serum calcium levels remained within normal ranges.

**Table 3:** False-positive events listed in NIRAF group.

Case	Gender	Age	BMI (kg/m^2^)	Resection scope	PTH (pg/mL)			Serum calcium (mmol/L)			Pathological diagnosis	Biopsy detected tissue
					Pre	Post D1	Post M1	Pre	Post D1	Post M1		
1	Female	33	22.58	Bilateral	21.70	27.60	32.50	2.34	2.11	2.14	Nodular goiter	Thyroid Nodule
2	Male	41	28.40	Unilateral	54.40	42.80	60.90	2.31	2.27	2.30	Thyroid carcinoma	Thymus
3	Male	30	33.22	Bilateral	44.40	24.00	40.68	2.39	2.16	2.37	Thyroid carcinoma	Adipose Tissue
4	Female	52	23.47	Unilateral	37.20	15.80	26.19	2.23	2.22	2.43	Thyroid carcinoma	Lymph Node

Abbreviations: BMI: body mass index; Post D1, the first day after surgery; Post M1, the first month after surgery; Pre, before the surgery; PTH: parathyroid hormone.

## Discussion

Although thyroidectomy is a well-established procedure, it carries inherent risks of postoperative complications. Postoperative hypocalcemia is the most common one [21]. Managing such complications requires accurate intraoperative identification of anatomical structures and prompt postoperative interventions, such as calcium and vitamin D supplementation [21]. Furthermore, while recent literature demonstrates that total thyroidectomy (TT) for differentiated thyroid carcinoma does not significantly increase the risk of early complications like hypoparathyroidism compared to subtotal thyroidectomy (STT), the overall incidence remains a substantial clinical challenge regardless of the surgical extent [22].

NIRAF has emerged as a transformative tool in endocrine surgery, significantly enhancing the intraoperative identification and preservation of PGs compared to traditional subjective visual inspection, and reducing the incidence of postoperative hypoparathyroidism [9]. For instance, a multicenter RCT demonstrated that NIRAF significantly increased the identification rate (47.1% vs. 19.2%, *P* < 0.001) and markedly reduced the risk of postoperative hypocalcemia (9.1% vs. 21.7%, *P* = 0.007) [5]. Our findings strongly confirm this clinical value. The NIRAF group demonstrated significantly higher postoperative PTH levels (*P* = 0.002) and serum calcium levels (*P* = 0.031) than those in the non-NIRAF group. This aligns with recent high-quality evidence, including comprehensive meta-analyses and multicenter randomized clinical trials (RCTs) [5], [7], [16]. Ultimately, NIRAF serves as a highly precise navigation tool that provides real-time guidance, translating into superior parathyroid preservation and improved biochemical stability for patients.

Despite its proven clinical benefits and high overall accuracy, the diagnostic precision of NIRAF is unsatisfactory, even in high-volume centers. A clinical trial by Kiernan et al. reported a false-positive rate of 11.1% due to overlapping intrinsic fluorescence from adjacent structures [11]. Similarly, another prospective study in 2023 reported a false-negative rate of 19.6% using NIRAF [23]. In our NIRAF group, we observed a false-negative rate of 20% and a false-positive rate of 16%, indicating that anatomical and optical interferences can still lead to inadvertent parathyroidectomy. Interestingly, our comparison revealed that the rate of inadvertent parathyroidectomy in the NIRAF group was not significantly different from that in the Non-NIRAF group (20% vs. 32%, *P* > 0.05). This suggests that false negatives do not inevitably contribute to inadvertent excision. To definitively minimize the inadvertent parathyroidectomy rate, Kuo TC et al. pointed that rather than relying exclusively on NIRAF, combining autofluorescence with adjunctive methods, such as PTH test strips or staining techniques, may be necessary to overcome these diagnostic limitations [24]. Therefore, to optimize the clinical application of this technology, a detailed investigation into the specific biological and technical causes of these inaccuracies is warranted.

The identification procedure for NIRAF is affected by false-negative signals. In our multivariate analysis, a higher BMI was associated with an increased incidence of inadvertent parathyroidectomy (*P* = 0.069). This assumption has several significant clinical implications. Excessive cervical fat accumulation substantially increases the risk of false-negative outcomes. Thick adipose tissue restricts the penetration depth of autofluorescence signals by altering light scattering [23]. Previous studies have reported that the maximum detectable depth of NIRAF reaches up to 3.05 mm [23]. Consequently, the inherently weak fluorescence emitted by true PGs enveloped in deep adipose tissue is easily obscured or attenuated, rendering them invisible on the display and ultimately increasing the risk of inadvertent excision during surgery [25], [26].

False-positive signals originating from surrounding nontarget tissues also impair diagnostic specificity and cause visual confusion. Our study documented four representative false-positive tissues, including thyroid nodules, adipose tissue, lymph nodes, and thymic tissue (Fig. 1). These interferences stem from the overlapping intrinsic optical and biological properties of these tissues. First, hyperplastic thyroid nodules exhibit abnormally enhanced autofluorescence, known as the “white light effect,” which is primarily associated with their internal colloid content and local vascular proliferation [18], [27], [28], [29]. Second, specific cervical adipose tissues contain intrinsic fluorophores that generate bright visual artifacts within the surgical field [30]. Third, reactive or metastatic lymph nodes present an endogenous optical signature similar to that of PGs, driven by their increased metabolic activity and nonspecific retention of fluorophores [18], [27], [31]. Finally, thymic tissue can exhibit confounding false-positive autofluorescence. Anatomically and embryologically, the thymus shares a common origin with inferior PGs, which may contribute to highly similar intrinsic fluorophore compositions that mislead visual assessment [32].

The hardware configuration, specifically probe-based versus camera-based systems, has been observed to have varying effects on the occurrence of both false positives and false negatives during parathyroid identification. Regarding probe-based systems, existing data indicate high sensitivity but limited impact on functional preservation. A 2025 multicenter RCT by Cousart et al. demonstrated improved PG identification (3.3 vs 2.8, *P* < 0.001) without significant difference in postoperative hypoparathyroidism rates [7]. Furthermore, this probe system significantly reduced the reliance on frozen section biopsies (4.0% vs. 11.2%, *P* = 0.01), thereby reducing false-positive excisions [7]. In contrast, camera-based imaging systems, which were utilized in our study, exhibited slightly different clinical outcomes. Benmiloud et al. reported that global spatial visualization provided by the camera-based system effectively mitigated inadvertent parathyroidectomy. Their multicenter RCT reported that this system significantly reduced inadvertent parathyroidectomy (2.5% vs. 11.7%, *P* = 0.006) [5]. A 2025 meta-analysis showed that the estimated overall accuracy of image-based methods is 0.96 (95% CI, 0.87–0.99), while the estimated overall accuracy of probe-based methods is 0.93 (95% CI, 0.92–0.94) [33]. The statistical significance was 0.36, indicating that there is insufficient evidence to indicate a significant difference in overall accuracy, sensitivity, and specificity between these two methods [33]. However, this meta-analysis also indicated that camera-based device offers distinct advantages in contactless global visualization, potentially favoring functional preservation, though direct comparative high-level evidence remains limited [33]. Some researches indicated that utilizing a 760–770 nm laser wavelength may be more conducive to improving diagnostic accuracy in camera-based systems [34], [35]. Moreover, the energy platforms utilized, such as the harmonic scalpel (HS) employed in our study, plays a crucial role in optimizing NIRAF accuracy. While both HS and LigaSure (LS) exhibit identical safety profiles regarding major bleeding, HS is significantly more effective at reducing minor bleeding, particularly in thyroid carcinoma (*P* = 0.02), according to the previous study [36]. This superior hemostatic performance minimizes blood exudation, thereby preventing false negatives caused by obscuration of the surgical field; meanwhile, compared to LS, HS features a finer tip that better limits thermal damage to surrounding tissue, consequently reducing nonspecific autofluorescence and false-positive visual interference. Future prospective comparative studies are required to definitively determine the optimal technological approach.

This study has several limitations. First, although we attempted to minimize bias through strict 1:1 matching of cases, this single-center design remains subject to potential selection bias. Second, the relatively small sample size (*n* = 50) limits the statistical significance of the multivariate analyses. It is necessary to verify secondary trends in a larger cohort. Finally, long-term complications were not fully assessed because of the short-term follow-up. To validate our conclusions, future prospective multicenter studies are essential to validate these findings.

In summary, this study confirmed that the NIRAF is a highly sensitive navigation tool that improves PG preservation and postoperative outcomes. However, its diagnostic accuracy is challenged by false positives from heterogeneous tissues and false negatives driven by anatomical barriers such as excessive adipose tissue. To address these challenges and enhance overall precision, future strategies could consider integrating multimodal platforms and optimizing surgical workflows through standardized protocols, thereby further elevating the safety of endoscopic thyroid surgery [31], [37], [38].

## CRediT authorship contribution statement

**Lihua Ao:** Writing – review & editing, Writing – original draft, Visualization, Methodology, Investigation, Formal analysis, Data curation, Conceptualization. **Yilin Li:** Writing – review & editing, Writing – original draft, Visualization, Validation, Methodology, Investigation, Formal analysis, Data curation, Conceptualization. **Jiaqi Cen:** Writing – review & editing, Visualization, Investigation, Formal analysis, Data curation. **Huiyuan Tang:** Writing – review & editing, Investigation, Data curation. **Wanyu Sun:** Writing – review & editing, Investigation, Data curation. **Zifeng Luo:** Writing – review & editing, Investigation, Formal analysis, Data curation. **Chang Cai:** Writing – review & editing, Validation, Supervision, Resources, Project administration, Methodology, Funding acquisition, Conceptualization. **Song Wang:** Writing – review & editing, Validation, Supervision, Resources, Project administration, Methodology, Funding acquisition, Conceptualization.

## Ethics approval

This study was approved by the Ethics Review Committee of the Fifth Affiliated Hospital of Guangzhou Medical University (ethics review approval number: GYWY-L2023-77).

## Funding sources

This work was supported by: the Ministry of Education Industry-University Cooperation Collaborative Education Project (No. 230902331264216); Guangdong Provincial Education Science Planning Project (Higher Education Special Project (No. 2025GXJK0131); Tertiary Education Scientific research project of Guangzhou Municipal Education Bureau (No. 2024312260); Clinical key specialty construction project funding of Guangdong Province (Guangdong Health Medical Letter [2023] No. 2); 10.13039/501100013254National College Student Innovation and Entrepreneurship Training Project (Key project) (No. 202610570001); 10.13039/100009659Guangzhou Medical University Student Innovation Ability Enhancement Program Project (No. 20261140).

## Declaration of competing interest

The authors declare that they have no known competing financial interests or personal relationships that could have appeared to influence the work reported in this paper.

## Data Availability

The data that support the findings of this study are available from the corresponding authors, Song Wang (gzwangmomo1983@sina.com) and Chang Cai (chanscai@163.com), upon reasonable request.
